# Developing students’ teaching through peer observation and feedback

**DOI:** 10.1007/s40037-015-0213-9

**Published:** 2015-09-10

**Authors:** Eliot L. Rees, Benjamin Davies, Michael Eastwood

**Affiliations:** School of Medicine, Keele University, North Staffordshire, ST5 5BG Keele, UK

**Keywords:** Medical education, Feedback, Peer teaching, Undergraduate

## Abstract

With the increasing popularity and scale of peer teaching, it is imperative to develop methods that ensure the quality of teaching provided by undergraduate students. We used an established faculty development and quality assurance process in a novel context: peer observation of teaching for undergraduate peer tutors. We have developed a form to record observations and aid the facilitation of feedback. In addition, experienced peer tutors have been trained to observe peer-taught sessions and provide tutors with verbal and written feedback. We have found peer observation of teaching to be a feasible and acceptable process for improving quality of teaching provided by undergraduate medical students. However, feedback regarding the quality of peer observer’s feedback may help to develop students’ abilities further.

## Background

Peer teaching is becoming an increasingly important part of undergraduate medical education [1]. It is internationally recognized that teaching skills should be developed at an early stage. This is reflected in expected graduate outcomes, for example the General Medical Council’s *Tomorrow’s Doctors* in the United Kingdom and the Liaison Committee on Medical Education in the United States [2, 3].

Given the recent rise in popularity of peer teaching [4], and the mandate for students to develop teaching skills [2, 3], a number of teaching skills development programmes have been created for medical students [5]. Such programmes, aimed at improving the quality of teaching provided by students, have been classified broadly into three approaches: learning through experience of peer teaching, teacher training workshops, and students teaching school pupils in outreach events [5]. Another method for improving the quality of teaching, which builds on learning from experience through peer teaching, is Peer Observation of Teaching (POT).

POT is a process in which an observer watches a colleague’s teaching and provides descriptive feedback on his/her teaching practice [6]. The process originated in higher education and has traditionally been used for observing faculty members, rather than students [7].

Gosling describes three models of POT: an evaluation model, a developmental model, and a collaborative model [8]. In the evaluation model, senior staff review juniors’ teaching in order to make a judgement on performance and to inform considerations regarding promotion. In the developmental model, expert educators observe and provide feedback in order to develop the observed teacher’s competency. Finally, in the collaborative model, peers observe each other’s teaching and provide feedback to stimulate self and mutual reflection and improve teaching practice.

Though the use of POT is growing in popularity [7], there has been limited research within the field of medical education [9–14], and none investigating its use with medical students. In one study, 20 clinicians involved in delivering a paediatrics module valued peer feedback, especially when delivered immediately after teaching. They also perceived the feedback to promote reflection, enhance teaching quality and be non-threatening [9]. In another study that surveyed 24 residents, 62 % had made conscious changes to their teaching in light of feedback and 57 % felt that the process made them better teachers [10]. These findings suggest that feedback on teaching can be useful for developing the competencies of novice teachers.

This paper aims to describe how we have introduced POT in peer teaching in undergraduate medical education at one school in the United Kingdom.

## Context

Keele University Medical School is a small (approximately 130 students per year) modern school with a highly integrated, hybrid, spiral curriculum. The school has a strong culture of extracurricular peer teaching that is autonomous from staff input. The vast majority of peer teaching within the school is delivered under the auspices of Keele Medical Education Society (KMES), a student-run society with the primary aim of facilitating high-quality peer teaching. Students in each year group deliver teaching to students in the academic year below them covering a variety of topics. Sessions are typically between one and two hours long, and take place on average fortnightly. Frequent topics taught include clinical examination, interpretation of investigations, anatomy and physiology, pathology, management of acute scenarios, and exam revision.

Over recent years, the scale of peer teaching has surged with most sessions attended by 10 to 40 students and the most popular events attracting up to 100 students per year group. Naturally, with extracurricular teaching of this magnitude, there were concerns about the quality of teaching provided by novice teachers.

In order to ensure the quality of teaching delivered by students to their peers is maximized, a programme of development opportunities has been introduced for students interested in teaching, including: peer-tutor training workshops focusing on lesson design and planning, peer review of lesson plans, and most recently, peer observation of teaching. In addition to quality assurance, POT was adopted as a process for developing students’ teaching skills.

## Peer observation of teaching

KMES have adopted a model, incorporating elements of both the developmental model and the collaborative model. The aim of POT within KMES is to develop both the tutor’s and the observer’s teaching practice through feedback, discussion and reflection. A collaborative approach emphasizes the mutual benefit to both tutor and observer. It is recognized that POT may evoke anxiety or apprehension in some tutors [8] and it is, therefore, emphasized that the purpose is developmental, supported by non-judgmental constructive dialogue, rather than a form of assessment [4]. In line with the developmental model, an action plan for improving teaching is generated through discussion between the tutor and observer.

POT was introduced in the academic year 2013–2014 and consisted of three stages: pre-observation meeting, observation of teaching, and post-observation feedback.

### Pre-observation meeting

Before teaching is due to take place, the observer and tutor meet to discuss the learning objectives for the session and set ground rules for the observation. As the feedback is delivered using the agenda-led model for feedback, tutors are encouraged to identify any particular areas they would like the observer to focus on during the observation.

Prior to the pre-observation meeting, the tutor is asked to forward a copy of the lesson plan and any resources to be used in order for observers to familiarize themselves.

### Observation of teaching

At the beginning of each session, the observer is introduced to the learners by the peer tutor. The observation then takes place for the entire duration of the session during which the observer makes descriptive notes to inform feedback. Observers were advised not to interrupt the sessions unless tutors sought their input.

### Post-observation feedback

Following the session, the tutor and observer meet to discuss the session whereby feedback is delivered through use of Pendleton’s rules [15], focusing on students’ specified areas for feedback. Subsequently, the observer facilitates the tutor in the generation of goals for personal development.

## Implementation

A form was developed so that observers could detail their comments and feedback. The form included sections for pre-observation, observation and post-observation. The pre-observation section consisted of areas for detailing intended learning outcomes for the session and preferred areas for feedback as identified by the tutor. The observation section included statements to be rated on a Likert scale and corresponding free-text areas for detailing strengths and areas for development. The post-observation section included a description of the overall quality of the session and an area identifying goals for future development.

During the first year of implementation, 11 observations were performed, each with one observer. The observers were not specifically trained to observe teaching and provide feedback, many felt under-prepared to give feedback to peers on their teaching. Furthermore, sufficient time was not always dedicated for pre- and post-observation meetings.

As such, a number of changes were incorporated into the process for the following academic year. These included: providing training for peer observers, amending the form, increasing the number of observations conducted, and encouraging adequate communication between observer and tutor prior to the observation, ensuring sufficient time allocation before and after the session.

The most fundamental change was the introduction of training for peer observers. The training consisted of a three-hour workshop designed and delivered by ER and BD to students with at least 1 year’s experience of peer teaching. The workshop discussed the principles and process of POT, principles of feedback, models of feedback delivery, and common pitfalls in feedback. Participants produced written feedback on example lesson plans and discussed their feedback in small groups. Participants were then introduced to KMES’ POT form and discussed how to complete it during observations. Participants subsequently watched a 10-minute video clip of an example peer teaching session during which they each completed a form. Following the clip, the group discussed the feedback from their observations, and one volunteer provided oral feedback during role-play with the tutor. This process was then repeated with a second example peer-teaching session video.

In addition to the provision of observer training, the form was also updated to include space for tutors to document their reflections on their teaching.

In the first semester of this academic year (2014–2015), 26 extracurricular peer-taught lessons were observed with the tutors receiving feedback. This is evidence of the feasibility of this form of tutor development and quality assurance activity. When contacted in advance regarding POT, no tutors have yet to decline to participate, suggesting it is acceptable to undergraduate student tutors.

## Further work

To further support peer-observer development, observers are offered the opportunity to receive feedback on their feedback in a process we have termed *Peer Review of Observation Feedback* (PROF). PROF involves senior students evaluating the written and/or verbal feedback provided by observers. To date, the use of PROF has been limited as the peer-observation programme is still a relatively new process within the school and the majority of resources have been focused on developing this programme first. However, further implementation of PROF would aid the development of peer observers and will be a focus in future years.

At present, there is no literature describing the effectiveness of peer observation of medical students’ teaching, exploring the quality of the feedback provided by peers, how student tutors use feedback on their teaching, and how this supports their development as teachers would enable us to better understand the value of peer observation of student teaching.

## Conclusions

This paper has described the use of an established faculty development and quality assurance process in a novel context: peer observation of teaching for undergraduate peer tutors.

### Funding

None.

### Other disclosures

None.

### Ethical approval

Not required.
